# Real-time analgesic efficacy and factors determining drug requirements of combined spinal-epidural analgesia for labor: a prospective cohort study

**DOI:** 10.1007/s00540-024-03368-8

**Published:** 2024-07-05

**Authors:** Shuzhen Wu, Yaxin Lu, Zijing Zhang, Linjia Zhong, Hongfei Dai, Changping Fang, Minli Huang, Zifeng Liu, Lingling Wu

**Affiliations:** 1 The Department of Obstetrics and Gynecology, The Third Affiliated Hospital Sun Yat-Sen University, No. 600 Tianhe Road, Tianhe District Guangzhou, Guangdong, China; 2 Big Data and Artificial Intelligence Center, The Third Affiliated Hospital Sun Yat-Sen University, Guangzhou, Guangdong, 510630 China

**Keywords:** Analgesic effectiveness, Anesthetic drug, Dosage adjustment, Labor analgesia, Labor monitoring

## Abstract

**Purpose:**

Combined spinal-epidural analgesia (CSEA) is effective but not sufficient for labor pain. This study was conducted to assess the real-time analgesic efficacy, side effects of anesthetic drug dosage, and maternal satisfaction in labor to provide reference for the optimization of labor analgesia.

**Methods:**

This was a prospective, cohort, single-center study that included 3020 women who received CSEA for labor analgesia. The visual analogue scale (VAS) for labor pain, real-time anesthetic drug dosage, side effects, adverse labor outcomes, factors influencing average drug dosage, and maternal satisfaction with CSEA were assessed.

**Results:**

Overall, the VAS labor pain score was lowest at the first hour after the anesthesia was given. After 4 h for primiparas and 3 h for multiparas, the VAS score was greater than 3 but the anesthetic drug dosage did not reach the maximum allowed dosage at the same time. The average anesthetic drug dosage was positively correlated with fever, urinary retention, uterine atony, prolonged active phase, prolonged second stage, assisted vaginal delivery, and postpartum hemorrhage. The average anesthetic drug dosage was the highest in women ≤ 20 years old, those with a body mass index (BMI) ≥ 24.9 kg/m^2^, and those with a primary or secondary education level.

**Conclusion:**

Appropriate age guidance and emphasis on education of labor analgesia, weight management during pregnancy, and real-time anesthetic dosage adjustment during labor based on VAS pain score may have positive effects on the satisfaction of labor analgesia.

**Clinical trial number and registry:**

Clinicaltrials.gov (ChiCTR2100051809).

## Introduction

Labor pain can result in negative effects such as dystocia and fetal distress [1]. Labor analgesia can reduce pain and thus the chances of negative effects [2]. Combined spinal-epidural anesthesia (CSEA) can rapidly relieve pain and its analgesic effect was more significant compared with other non-neuraxial analgesic protocols [3, 4], and furthermore, it can reduce the rate of non-medically indicated cesarean deliveries [5, 6]. However, studies have reported that as analgesic time is prolonged the visual analogue scale (VAS) score for pain during labor gradually rises, suggesting that the relief of labor pain becomes insufficient [7, 8]. The specific changes of labor pain and analgesic demand during labor remain unclear because few studies have evaluated time-course changes in effectiveness of analgesia during labor [9].

Jib et al. dynamically assessed pain in adolescents with cancer and provided real-time pain treatment to decrease the intensity of pain [10]. A recent study evaluated the pain scores of women using ibuprofen and oxycodone/acetaminophen for medical abortion pain relief at regular intervals, showing more pain relief and lower use of analgesic agents [11]. These two results suggested that frequent assessment of pain can be used as the basis for pain treatment. Ban et al. adjusted the background drug infusion every hour based on the anesthetic requirement in the previous 1 h for improving maternal analgesia satisfaction in their study of computer-integrated patient-controlled epidural analgesia [12], suggesting that pain assessment and adjustment of analgesia every 1 h is a feasible approach when establishing precise and effective analgesic protocols.

Sufentanil combined with ropivacaine alleviates labor pain significantly, but is associated with dose-dependent side effects such as fever, urinary retention, and uterine atony [13–15]. Studies have shown that drug dose prediction based on influencing factors can improve medication safety [16, 17]. Thus, the effectiveness and safety of analgesics should be always considered together and a fine balance must be searched for to achieve optimal labor analgesia. Studies of this nature, however, have not been performed to examine pain scores, drug dosages, and factors influencing drug dosage for women in labor.

In addition, it has been reported that parity is a factor that influences labor pain and the management standards for primipara and multipara in the new stage of labor are not consistent [18, 19].

To provide reference for the optimization of labor analgesia in the primiparas and multiparas, this study was conducted to assess the time-course of VAS scores during labor within a pre-set CSEA protocol (drug doses included), side effects of anesthetic drug dosage, and maternal satisfaction.

## Materials and methods

### Study design

This study was a prospective, single-center observational cohort study conducted at the Third Affiliated Hospital of Sun Yat-sen University, an institution with 7,000 deliveries per year and a labor analgesia rate of more than 50%. A total of 3,020 women who delivered from July 2020 to January 2022 were included in the study. They were divided into two groups: primiparas and multiparas. Inclusion criteria included: (1) Singleton pregnancy; (2) Accepted CSEA with PCEA for labor analgesia; (3) No contraindications to the analgesics used or method of administration; (4) Cervical dilation of 2 cm before analgesia was administered; (5) No mental illness. Exclusion criteria included: (1) Twin pregnancies; (2) Breech presentation; (3) Contraindications for vaginal delivery; (4) Combination use of other analgesic methods.

This study was registered at Clinicaltrials.gov, and given the registration number ChiCTR2100051809. The study was approved by the Ethics Committee of the Third Affiliated Hospital of Sun Yat-Sen University (No. [2022] 02–051-01), and all participants provided written informed consent.

### Analgesic procedure

According to the relevant literature and guidelines, the program of labor analgesia formulated by the Department of Anesthesiology of this research institution was as follows [3, 13]. With the woman in a lateral decubitus position, 5 μg sufentanil was injected into the subarachnoid space at L_2-3_, and an epidural catheter attached to the PCEA pump was inserted into the epidural space. Then, a PCEA pump containing a 120 ml solution of 45 µg sufentanil (Yichang Renfu Pharmaceutical Co., Ltd) and 75 mg ropivacaine (AstraZeneca) was started. The pump provided a continuous infusion with a background infusion dose (D_bi_) of 6 ml/h, a bolus dosage (D_b_) of 8 ml, and a lock-out time interval of 15 min. The maximum dosage (D_m_) of PCEA pump was 38 ml/h (containing 14.25 µg sufentanil and 23.75 mg ropivacaine). The pump was used until the completion of perineal suturing. Effective analgesia was considered a VAS pain score of ≤ 3. If pain relief was inadequate (VAS score > 3), a supplementary bolus dose of 8 ml was given [1]. The number of supplementary bolus (N_s_) of the PCEA pump per hour, total anesthetic drug dosage (D_t_) used, and the time of labor analgesia duration (t) were recorded during labor. Based on the 2019 American College of Obstetrics and Gynecology (ACOG) recommendations and drug instructions for the safe dosage of sufentanil and ropivacaine (45 μg/h and 28 mg/h respectively) [3], the safe dosage (D_s_) of the PCEA pump was calculated. To ensure the accuracy of data, all the subjects received research education.

### Data collection

A VAS pain score (with a VAS-scale printed in a paper) was used to assess labor pain, with 0 = no pain and 10 = pain as bad as it could be [20, 21]. In this study, scores were categorized as 0 = no pain; 1–3 = mild pain; 4–6 = moderate pain; and 7–10 = severe pain [7]. VAS pain scores were recorded before labor analgesia, after labor analgesia that was begun at 10 min, 30 min, and hourly from 1 to 10 h, at full cervical dilatation, and at 1, 2, 3, and 4 h after full cervical dilatation. Because this was an observational study, no additional analgesic dose would be added despite results of real-time assessment indicated that such a dose would be needed. To ensure the accuracy and reliability of data, all the physicians received standardized training.

The primary study variable was the VAS pain score. Data collected include the real-time anesthetic dosage, average anesthetic dosage, the occurrence of side effects including fever, urinary retention, uterine atony, and prolonged fetal heart rate (FHR) deceleration, and adverse labor outcomes including prolonged labor phase, assisted vaginal delivery, postpartum hemorrhage, and neonatal asphyxia.The complications related to CSEA such as epidural hematoma, motor block, accidental dural puncture (ADP) and post-dural puncture headache (PDPH) were also documented. Maternal satisfaction was recorded based on a scale of 1 to 5: 1 = very dissatisfied; 2 = dissatisfied; 3 = fair; 4 = satisfied; and 5 = very satisfied [22].

The real-time dosage of the PCEA pump (D_r_) was considered to indicate the real-time anesthetic drug consumption from the PCEA pump during labor per hour. The formula for calculating D_r_ was: D_r_ = D_bi_ + N_s_ × 8. The average dosage (D_a_) of PCEA pump was calculated by the following formula: D_a_ = D_t_ /t.

### Statistical analysis

Double-entry data and consistency checks were used (Epidata 3.0) [23]. Data were analyzed using IBM SPSS statistical software and SAS software. Quantitative variables were reported as mean ± standard deviation (SD), and compared by t-test or Wilcoxon rank-sum test. Categorical variables were reported as count and percentage, and compared by the chi-square test or Fisher’s exact test. The reference range of labor anesthesia duration was estimated by the 95% confidence interval (CI). Generalized estimating equation (GEE) analysis was used to analyze the repeated measures (VAS pain score and real-time anesthetic dosage). The correlation between the average anesthetic dosage and side effects or adverse labor outcomes was examined using logistic regression analysis. Spearman’s correlation analysis was used to analyze factors influencing average anesthetic drug dosage. Linear regression analysis was used for assessing factors influencing the average drug dosage after adjustment for multiple factors. The level of significance was set at α = 0.05.

## Results

### Study participants

**Fig. 1:**
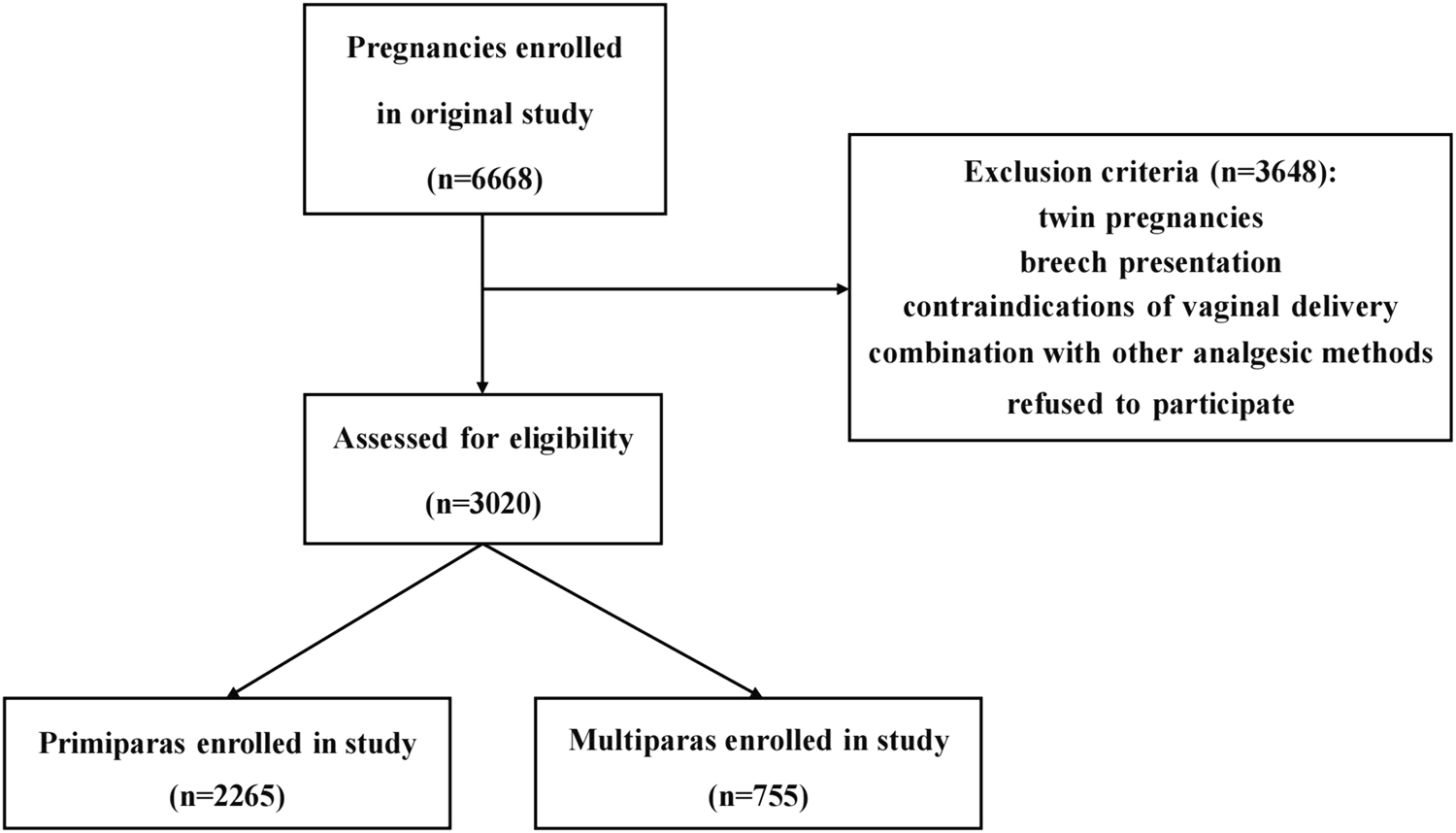
Patient eligibility

A total of 3,020 women participated in this study, among which there were 2,265 primiparas and 755 multiparas (Fig. 1). The clinical information of the 2 groups and *P* values for assessing group differences are shown in Table 1. The epidural hematoma and motor block did not occur because of the experienced anesthesiologist and adequate preparation. There were 3 and 1 ADP cases in primiparas and multiparas respectively, but no PDPH cases were observed in both groups.

**Table 1:** Comparison of clinical information for primiparas and multiparas

Term	Primiparas (n = 2265)	Multiparas (n = 755)	*P* value
*Baseline data*			
Age (year)	28.7 ± 3.1	32.0 ± 3.8	< 0.01*
Gestational week (week)	39.3 ± 1.3	39.3 ± 1.1	NS
Prenatal BMI (kg/m^2^)	21.2 ± 3.3	21.6 ± 3.2	NS
Newborn Weight (kg)	3.0 ± 0.3	3.2 ± 0.3	0.05*
Education level			NS
Primary/Secondary	88 (3.8)	36 (4.7)	
High School Education	135 (5.9)	55 (7.2)	
College/University (less than 4 years)	914 (40.3)	293 (38.8)	
College/University (4 years and above)	1128 (49.8)	371 (49.1)	
*Clinical data*			
The way for induction			NS
Combined cervical dilation balloons with contraction	245 (10.8)	76 (10.0)	
Cervical dilation balloons	38 (1.7)	11 (1.4)	
Small doses of contractions	207 (9.1)	73 (9.7)	
None	1775 (78.3)	595 (78.7)	
*Labor duration (hour)*			
Latent phase	7.1 ± 2.7	4.8 ± 2.4	< 0.01*
Active phase	3.6 ± 1.4	2.4 ± 1.1	< 0.01*
Second stage	1.2 ± 0.8	0.4 ± 0.2	< 0.01*
The duration of effective analgesia (hour)	4.2 ± 1.2	3.8 ± 1.1	< 0.05*
The duration of labor analgesia (hour)	(8.1–8.6)	(4.0–4.3)	< 0.05*
Average anesthetic drug dosage (ml/hour)	10.0 ± 5.0	9.2 ± 5.0	< 0.01*
*Side effect*			
Fever	622 (27.5)	103 (13.6)	< 0.01*
Urinary retention	900 (39.7)	204 (27.0)	< 0.01*
Uterine atony	994 (43.9)	159 (21.1)	< 0.01*
Prolonged FHR deceleration	349 (15.4)	77 (10.2)	< 0.01*
*Adverse labor outcome*			
Prolonged latent phase	211 (9.3)	69 (9.1)	NS
Prolonged active phase	134 (5.9)	28 (3.7)	0.02*
Prolonged second stage of labor	78 (3.4)	15 (1.9)	0.04*
Vaginal assisted birth	156 (6.9)	18 (2.4)	< 0.01*
Postpartum hemorrhage	274 (12.0)	66 (8.7)	0.01*
*Complications*			
Epidural hematoma	0(0.0)	0(0.0)	NS
Motor block	0(0.0)	0(0.0)	NS
Accidental dural puncture	3(0.0)	1(0.0)	NS
Post-dural puncture headache	0(0.0)	0(0.0)	NS

Data are presented as mean ± SD or count (%)

BMI body mass index, FHR fetal heart rate

An orifice dilatation of ≥ 6 cm was used as a marker of active stage [24]

*NS* as non-significant. *Evidence for an association assessed at *P* value ≤ 0.05

### VAS scores and anesthetic drug dosages

**Fig. 2:**
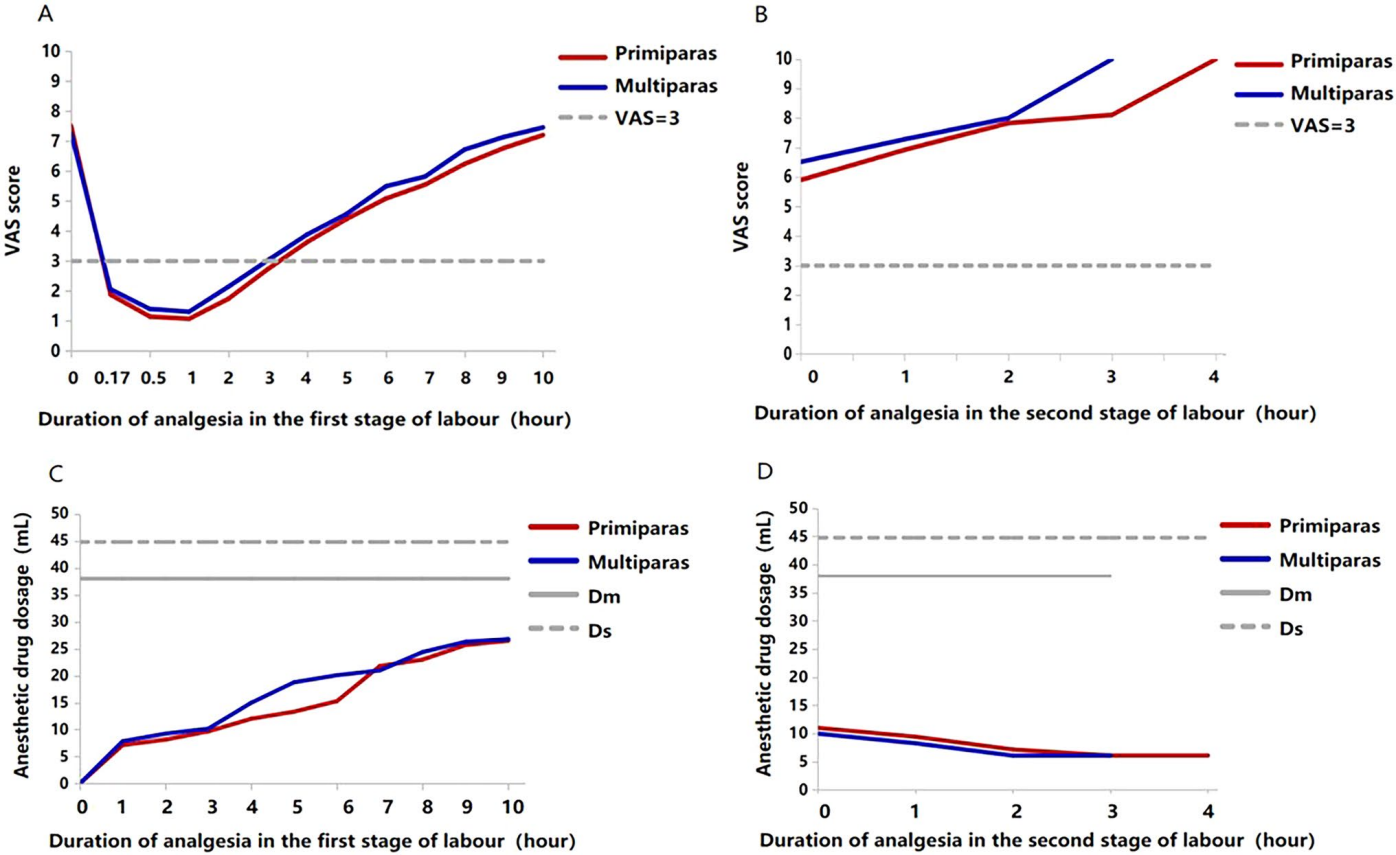
Comparison of analgesia efficacy between primiparas and multiparas at the same moment. The real-time VAS score with the duration of analgesia (**A**, **B**) and the real-time anesthetic drug dosage with the duration of analgesia (**C**, **D**). Both of the real-time VAS score and the real-time anesthetic drug dosage changed gradually over time (*P* < 0.05). D_m:_ the maximum dosage of PCEA pump. D_s:_ the safe dosage of the PCEA pump

As shown in Table 2, the VAS pain scores for primiparas and multiparas were lowest at the first hour after the anesthesia was given. The duration of effective analgesia for primiparas was 4.2 ± 1.2 h, and for multiparas was 3.8 ± 1.1 h, which were significantly shorter than the duration of labor analgesia (Table 1). After 4 h of analgesia for primiparas and 3 h of analgesia for multiparas, the VAS pain scores were > 3 for both groups (Fig. 2A, B).

**Table 2:** Real-time VAS pain score changes during labor with analgesia

Term	Primiparas		Multiparas	
	n	VAS	N	VAS
Before labor analgesia	2265	7.52 ± 1.90	755	7.27 ± 1.90
*After analgesia in the first stage of labor (hour)*				
0.17	2265	1.88 ± 1.92	755	2.06 ± 2.01
0.5	2265	1.15 ± 1.51	755	1.40 ± 1.72
1	2247	1.07 ± 1.44	712	1.31 ± 1.63
2	2068	1.74 ± 1.83	536	2.15 ± 2.03
3	1592	2.73 ± 2.11	339	3.02 ± 2.22
4	1260	3.63 ± 2.17	192	3.88 ± 2.35
5	930	4.40 ± 2.10	123	4.57 ± 2.37
6	692	5.08 ± 2.08	81	5.49 ± 2.06
7	507	5.55 ± 1.81	40	5.82 ± 2.03
8	362	6.24 ± 1.98	25	6.72 ± 1.95
9	258	6.77 ± 2.09	14	7.14 ± 1.99
10	189	7.21 ± 2.03	11	7.46 ± 1.81
*The second stage of labor (hour)*				
0	2265	5.90 ± 2.49	755	6.52 ± 2.41
1	965	6.92 ± 1.93	93	7.28 ± 2.20
2	335	7.83 ± 2.23	14	8.00 ± 2.83
3	89	8.11 ± 2.21	3	10.00 ± 0.00
4	27	10.00 ± 0.00	-	-

Data are presented as mean ± SD

However, after 4 h of analgesia for primiparas and 3 h of analgesia for multiparas, the anesthetic drug dosage was below the maximum dosage (D_m_) of the PCEA pump, and below the safe dosage (D_s_) of anesthetic drug (Fig. 2A, B). The average anesthetic dosage was higher in primiparas than in multiparas (*P* < 0.05, Table 1). The GEE analysis revealed that the anesthetic drug dosage increased in tandem with the VAS pain score during the first stage of labor for primiparas and multiparas (both, *P* < 0.05, Table 3).

**Table 3:** Analysis of real-time VAS pain score and anesthetic drug dosage after analgesia

Term	Primiparas				Multiparas			
	Beta	SE	95% CI	*P* value	Beta	SE	95% CI	*P* value
First stage	5.37	0.12	5.13–5.60	< 0.01	3.23	0.19	2.85–3.61	< 0.01
Second stage	0.03	0.02	– 0.01 to 0.08	0.19	0.02	0.07	0.06–0.08	0.21

### Side effects and adverse labor outcomes

**Fig. 3:**
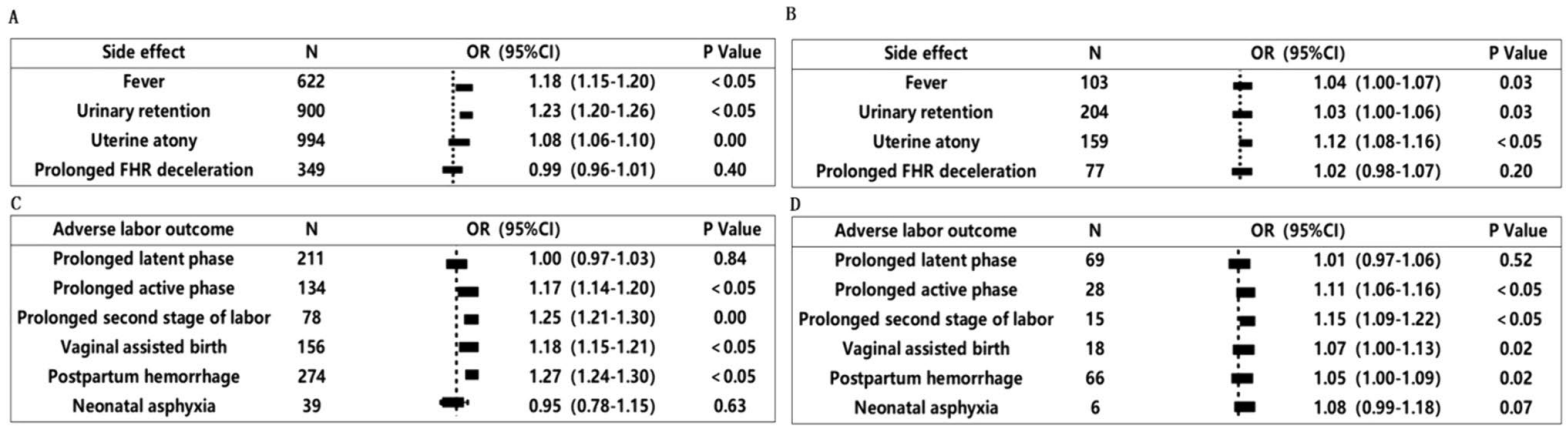
Correlation analysis of the average anesthetic dosage with the side effect (**A**, **B**) and adverse labor outcomes (**C**, **D**)

Logistic regression models were developed to determine if the average anesthetic drug dosage was positively associated with the incidence of side effects and adverse labor outcomes. For both primiparas and multiparas, the average drug dosage was positively correlated with fever, urinary retention, uterine atony, prolonged active phase, prolonged second stage, assisted vaginal delivery and postpartum hemorrhage (all, *P* < 0.05, Fig. 3A–D). The side effects of analgesia and the incidence of adverse labor outcomes were higher in primiparas than in multiparas (both, *P* < 0.05, Table 1).

### Univariate and multivariate analysis

Univariate analysis indicated that primipara age, prenatal BMI, newborn weight, and education level, as well as multipara age, prenatal BMI, and education level were all related to the average anesthetic drug dosage (all, *P* < 0.05, Table 4). Multivariate analysis with adjustment showed that the average anesthetic drug dosage for the 2 groups was influenced by age, prenatal BMI, and education level. In addition, the average anesthetic drug dosage was highest in women ≤ 20 years old, with a BMI ≥ 24.9 kg/m^2^, and with a primary or secondary education level (Table 4).

**Table 4:** Analysis of factors influencing the average anesthetic dosage

Term	Univariate analysis			Multivariate analysis		
	B	95% CI	*P* value	B	95% CI	*P* value
Primiparas						
Age (year)*						
≤ 20	– 0.722	– 1.164 to – 0.280	< 0.01	– 0.642	– 0.966 to – 0.317	< 0.01
20–30						
> 30						
Prenatal BMI (kg/m^2^)*						
≤ 18.5	– 0.704	– 1.055 to – 0.352	< 0.01	0.158	0.101–0.215	0.03
18.5–24.9						
≥ 24.9						
Newborn Weight (kg)	1.068	0.427–1.709	< 0.01	0.505	– 0.235 to 1.245	0.18
Education level*	– 6.448	– 6.679 to – 6.218	< 0.01	– 6.453	– 6.684 to – 6.222	< 0.01
Primary / Secondary						
High School Education						
College/University (less than 4 years)						
College/University (4 years and above)						
Multiparas						
Age (year)*						
≤ 20	– 5.439	– 6.080 to – 4.799	< 0.01	– 2.399	– 2.897 to – 1.900	< 0.01
20–30						
> 30						
Prenatal BMI (kg/m^2^)*						
≤ 18.5	5.589	5.090–6.088	< 0.01	2.247	1.760–2.734	< 0.01
18.5–24.9						
≥ 24.9						
Newborn Weight (kg)	−0.465	– 1.382 to 0.453	0.32	–	–	–
Education level*						
Primary / Secondary	– 3.606	– 3.997 to – 3.216	< 0.01	– 2.168	– 2.547 to – 1.790	< 0.01
High School Education						
College/University (less than 4 years)						
College/University (4 years and above)						

*Evidence for an association assessed at *P* value ≤ 0.05

### Maternal satisfaction

The proportion of multiparas (73.5%) who felt satisfied with labor analgesia was higher than the proportion of primiparas (67.3%). An analysis of the reasons for a satisfaction score ≤ 3 showed a statistical difference for primiparas and multiparas, and poor analgesia was the main cause (χ^2^ = 17.757). Further analysis showed that compared to multiparas, a smaller proportion of primiparas were unsatisfied with the poor analgesia (55.8%), but had a higher frequency of side effects (13.9%) and prolonged labor (27.1%) (Table 5).

**Table 5:** Satisfaction scores and reasons for unsatisfactory labor analgesia

Term	Primiparas	Multiparas	*P* value
Maternal satisfaction			< 0.01*
Satisfaction score ≥ 4	1525 (67.3)	555 (73.5)	
Satisfaction score ≤ 3	740 (32.6)	200 (26.4)	
Reasons for unsatisfactory labor analgesia			
Poor analgesia	413 (55.8)	124 (62.0)	< 0.01*
Prolonged labor	201 (27.1)	46 (23.0)	0.02*
Side effect	103 (13.9)	21 (10.5)	< 0.01*
Else (such as experience)	23 (3.1)	9 (4.5)	NS

Data are presented as count (%)

*NS* as non-significant. *Evidence for an association assessed at *P* value ≤ 0.05

The maternal satisfaction score ≥ 4 was considered satisfactory for labor analgesia

## Discussion

A study by Eran Ashwal in 2020 concluded that the cervical dilation rate during labor analgesia differed between primiparas and multiparas [25]. In the current study, the VAS pain scores were lowest at the first hour after the anesthesia was given, and indicated ineffective analgesia after 4 h for primiparas and 3 h for multiparas. There are a few numbers of reasons for this finding. Firstly, the best analgesic effect may be obtained at these times as a result of the pharmacological action or the method of administration of the anesthetic drug [26]. Secondly, physical exhaustion during labor might lead to a decreased tolerance of labor pain [27], and prolonged opioid usage can result in adaptive changes in μ receptors leading to tolerance and hyperalgesia [28]. The tolerance for labor pain reaches a limit after 4 h of analgesia for primiparas and 3 h of analgesia for multiparas. Thirdly, our results showed that the anesthetic drug dosage increased in tandem with the VAS pain score during the first stage of labor, but the maximum dosage of PCEA pump was not reached after 4 h for primiparas and 3 h for multiparas, prior to which the pump with a pre-set CSEA protocol may provide too much anesthetic drug dosage. After that, the PCEA pump was not fully used, reflecting the limitations of the PCEA pump settings and even the CSEA protocol.

It has been shown that multiparas require a higher median effective concentration of ropivacaine during labor analgesia due to psychogenic pain from previous labor pain experiences [29]. This is consistent with the satisfaction survey of this study which showed that compared to primiparas, more multiparas experienced poor labor analgesia. So, primiparas and multiparas may require different management and awareness of self-management should be given more attention. In our study, we also found that insufficient dosage was one of the factors leading to unsatisfactory labor analgesia for both primiparas and multiparas. Therefore, clinicians need to adjust the background infusion dose, the bolus dosage, or the lock-out time of PCEA pump timely under the safe dosage of sufentanil and ropivacaine (45 μg/h and 28 mg/h respectively [3]) and educate women that the drug dosage may be increased according to the real-time VAS pain score in order to provide more prolonged relief of pain after 4 h of analgesia for primiparas and 3 h of analgesia for multiparas [30, 31].

The incidence of fever is associated with the dose of anesthetic drugs, and fever associated with ropivacaine is due to the release of inflammatory factors caused by ropivacaine, while the use of sufentanil may result in impairment of the central thermoregulatory response [32, 33]. Armstrong et al. proposed that opioid-induced urinary retention is related to dosage, and may be the result of detrusor muscle dysfunction or because the drug impedes the afferent and efferent mechanisms of the voiding reflex [34]. The aforementioned studies help to explain our observation. It also showed that the average anesthetic drug dosage was associated with uterine atony, prolonged active phase, prolonged second stage, assisted vaginal delivery and postpartum hemorrhage. These findings could be related to a decrease in the frequency and intensity of uterine contractions, because the nerves innervating the uterus are inhibited by CSEA resulting in uterine atony. And uterine atony and assisted vaginal delivery are known as risk factors for postpartum hemorrhage [35–38]. Primiparas may have a higher average anesthetic drug dosage due to their longer duration of labor analgesia, and this may be why a higher proportion of primiparas were dissatisfied due to side effects and prolonged labor.

It has been shown that an increased BMI is linked to increased technical difficulties and failure of epidural analgesia for labor, resulting in insufficient analgesia [39]. Junge et al. reported a strong correlation between severe fear and labor pain. Younger women, who may feel more fear of childbirth due to a lack of education have a higher demand for analgesic drugs [40]. On the other hand, women with higher education levels have a higher awareness of analgesia and get more effective pain relief [31]. As revealed in this study, the average anesthetic drug dosage was the highest in the groups ≤ 20 years old, BMI ≥ 24.9 kg/m^2^, and those with a primary or secondary education level. The findings of our study also demonstrated that there was still potential for ropivacaine and sufentanil dosage adjustments of CSEA for labor analgesia. The parturients received relevant education before labor through various means, including network courses offered by our hospital, to alleviate anxiety and provide information on the use of anesthetics and their side effects. However, their awareness of the current CSEA protocol remained limited. As for the reasons of unsatisfactory labor analgesia, insufficient analgesia was the main cause, followed by side effects and prolonged labor, among which the primiparas and multiparas were different. So individualized labor analgesia programs based on real-time VAS pain score and parity could be establish (ex. multiparas want more analgesics, primiparas are more concerned about the side effects of labor analgesia, etc.). Furthermore, clinicians should strengthen weight management during pregnancy and educate patients regarding labor analgesia awareness to reduce the use of anesthetic drugs, in order to achieve the balance between sufficient analgesic and side effects of administered drugs during the whole course of labor.

There may be some possible limitations in this study. First, the selection of 1-h intervals for VAS pain scores during labor analgesia had the time constraint, which might be shortened to make the assessment more specific. Second, a future study should be conducted in multicenter study to validate the findings in our one-institution-based study. Third, only one CSEA protocol was researched in this study. And fourth, there were also possible confounding factors that have not been studied, including environmental factors, family factors and so on.

## Conclusion

Clinician should establish individualized labor analgesia programs based on real-time VAS pain score and parity. At the same time, education of labor analgesia should be strengthened to enhance the effectiveness of labor analgesia, reduce side effects, and improve maternal satisfaction.

## Data Availability

The data that support the findings of this study are available on request from the corresponding author, [LW], upon reasonable request.

## References

[1] Rooks JP. Labor pain management other than neuraxial: what do we know and where do we go next? Birth. 2012;39(4):318–22. 23281953 10.1111/birt.12009

[2] Yin H, Tong X, Huang H. Dural puncture epidural versus conventional epidural analgesia for labor: a systematic review and meta-analysis of randomized controlled studies. J Anesth. 2022;36(3):413–27. 35445869 10.1007/s00540-022-03061-8

[3] American College of Obstetricians and Gynecologists’ Committee on Practice Bulletins—Obstetrics. ACOG Practice Bulletin No. 209: Obstetric Analgesia and Anesthesia. Obstet Gynecol. 2019;133(3): e208-e225. 10.1097/AOG.0000000000003132 30801474

[4] Lam KK, Leung MKM, Irwin MG. Labour analgesia: update and literature review. Hong Kong Med J. 2020;26(5):413–20. 32943586 10.12809/hkmj208632

[5] Bonnet MP, Prunet C, Baillard C, Kpéa L, Blondel B, Le Ray C. Anesthetic and obstetrical factors associated with the effectiveness of epidural analgesia for labor pain relief: an observational population-based study. Reg Anesth Pain Med. 2017;42(1):109–16. 27831958 10.1097/AAP.0000000000000517

[6] Wang Q, Zheng SX, Ni YF, Lu YY, Zhang B, Lian QQ, Hu MP. The effect of labor epidural analgesia on maternal-fetal outcomes: a retrospective cohort study. Arch Gynecol Obstet. 2018;298(1):89–96. 29777348 10.1007/s00404-018-4777-6

[7] Sun X, Zhou Q, Zhou M, Cao R, Chen Z, Tang S, Huang S. The effect of epidural nalbuphine combined with ropivacaine on epidural analgesia during labor: a multicenter, randomized, double-blind, controlled study. Clin J Pain. 2021;37(6):437–42. 33758132 10.1097/AJP.0000000000000928

[8] Song Y, Du W, Zhou S, Zhou Y, Yu Y, Xu Z, Liu Z. Effect of dural puncture epidural technique combined with programmed intermittent epidural bolus on labor analgesia onset and maintenance: a randomized controlled trial. AnesthAnalg. 2021;132(4):971–8. 10.1213/ANE.0000000000004768 32282386

[9] Mao L, Zhang X, Zhu J. Analgesic effects, birth process, and prognosis of pregnant women in normal labor by epidural analgesia using sufentanil in combination with ropivacaine: a retrospective cohort study. ComputIntellNeurosci. 2022;2022:1228006. 10.1155/2022/1228006 PMC9444351 36072747

[10] Jibb L, Nathan PC, Breakey V, Fernandez C, Johnston D, Lewis V, McKillop S, Patel S, Sabapathy C, Strahlendorf C, Victor JC, Moretti ME, Nguyen C, Hundert A, Cassiani C, El-KhechenRichandi G, Insull H, Hamilton R, Fang G, Kuczynski S, Stinson J. Pain Squad+ smartphone app to support real-time pain treatment for adolescents with cancer: protocol for a randomised controlled trial. BMJ Open. 2020;10(3): e037251. 32184315 10.1136/bmjopen-2020-037251 PMC7076249

[11] Friedlander EB, Raidoo S, Soon R, Salcedo J, Davis J, Tschann M, Fontanilla T, Horiuchi W, Kaneshiro B. The experience of pain in real-time during medication abortion. Contraception. 2022;110:71–5. 35283083 10.1016/j.contraception.2022.03.003

[12] Sng BL, Woo D, Leong WL, Wang H, Assam PN, Sia AT. Comparison of computer-integrated patient-controlled epidural analgesia with no initial basal infusion versus moderate basal infusion for labor and delivery: a randomized controlled trial. J Anaesthesiol Clin Pharmacol. 2014;30(4):496–501. 25425774 10.4103/0970-9185.142842 PMC4234785

[13] Wang Y, Xu M. Comparison of ropivacaine combined with sufentanil for epidural anesthesia and spinal-epidural anesthesia in labor analgesia. BMC Anesthesiol. 2020;20(1):1. 31898488 10.1186/s12871-019-0855-y PMC6939327

[14] Anim-Somuah M, Smyth RM, Cyna AM, Cuthbert A. Epidural versus non-epidural or no analgesia for pain management in labour. Cochrane Database Syst Rev. 2018;5(5):CD000331. 10.1002/14651858.CD000331.pub4 PMC6494646 29781504

[15] Edwards IR, Aronson JK. Adverse drug reactions: definitions, diagnosis, and management. Lancet. 2000;356(9237):1255–9. 11072960 10.1016/S0140-6736(00)02799-9

[16] Salinas AS, Lorenzo-Romero J, Segura M, Calero MR, Hernández-Millán I, Martínez-Martín M, Virseda JA. Factors determining analgesic and sedative drug requirements during extracorporeal shock wave lithotripsy. Urol Int. 1999;63(2):92–101. 10592496 10.1159/000030425

[17] Hashimoto M, Aogaki K, Numata C, Moriwaki K, Matsuda Y, Ishii R, Tanaka I, Okamoto Y. Factors influencing the prescribed dose of opioid analgesics in cancer patients. J Opioid Manag. 2020;16(4):247–52. 32885832 10.5055/jom.2020.0578

[18] Westergren A, Edin K, Lindkvist M, Christianson M. Exploring the medicalisation of childbirth through women’s preferences for and use of pain relief. Women Birth. 2021;34(2):e118–27. 32094035 10.1016/j.wombi.2020.02.009

[19] Expert consensus on new labor standards and management [J]. Chinese J Obstet Gynecol 2014:49(07):486.

[20] Thorburn PT, Monteiro R, Chakladar A, Cochrane A, Roberts J. South East Anaesthetic Research Chain (SEARCH), Mark Harper C. Maternal temperature in emergency caesarean section (MATES): an observational multicentre study. Int J ObstetAnesth. 2021;46:102963. 10.1016/j.ijoa.2021.102963 33773300

[21] Thong ISK, Jensen MP, Miró J, Tan G. The validity of pain intensity measures: what do the NRS, VAS, VRS, and FPS-R measure? Scand J Pain. 2018;18(1):99–107. 29794282 10.1515/sjpain-2018-0012

[22] Wang Fen, LI Yan, Chen Feng-Ren, et al. Effect of maternal satisfaction with labor analgesia on postpartum depression. Modern Preventive Medicine, 2020;47(06):1028–1031+1051.

[23] Yue-song PAN. Data management and quality control for clinical research. Med J Pumch. 2018;9(5):458–62.

[24] Zhang J, Landy HJ, Ware Branch D, Burkman R, Haberman S, Gregory KD, Hatjis CG, Ramirez MM, Bailit JL, Gonzalez-Quintero VH, Hibbard JU, Hoffman MK, Kominiarek M, Learman LA, Van Veldhuisen P, Troendle J, Reddy UM. Consortium on Safe Labor. Contemporary patterns of spontaneous labor with normal neonatal outcomes. Obstet Gynecol. 2010;116(6):1281–1287. 10.1097/AOG.0b013e3181fdef6e PMC3660040 21099592

[25] Ashwal E, Livne MY, Benichou JIC, Unger R, Hiersch L, Aviram A, Mani A, Yogev Y. Contemporary patterns of labor in nulliparous and multiparous women. Am J Obstet Gynecol. 2020;222(3):267.e1-267.e9. 31574290 10.1016/j.ajog.2019.09.035

[26] Congedo E, Sgreccia M, De Cosmo G. New drugs for epidural analgesia. Curr Drug Targets. 2009;10(8):696–706. 19702518 10.2174/138945009788982441

[27] Siyoum M, Mekonnen S. Labor pain control and associated factors among women who gave birth at Leku primary hospital, southern Ethiopia. BMC Res Notes. 2019;12(1):619. 31547839 10.1186/s13104-019-4645-x PMC6757368

[28] Colvin LA, Bull F, Hales TG. Perioperative opioid analgesia-when is enough too much? A review of opioid-induced tolerance and hyperalgesia. Lancet. 2019;393(10180):1558–68. 30983591 10.1016/S0140-6736(19)30430-1

[29] Peng Q, Yang Z, Zhang W, Wu X. Comparison of median effective concentration of ropivacaine in multiparas or primiparas during epidural labor analgesia: STROBE compliant. Medicine (Baltimore). 2020;99(1): e18673. 31895835 10.1097/MD.0000000000018673 PMC6946190

[30] Gido R, Yadeta TA, Tura AK. Utilization of obstetric analgesia for labor pain management and associated factors among obstetric care providers in public hospitals of addis ababa, ethiopia: a cross-sectional study. ObstetGynecol Int. 2021;2021:9973001. 10.1155/2021/9973001 PMC8629664 34853595

[31] Ali Alahmari SS, ALmetrek M, Alzillaee AY, Hassan WJ, Ali Alamry SM. Knowledge, attitude, and practice of childbearing women toward epidural anesthesia during normal vaginal delivery in Alsanayeah Primary Health Care in Khamis Mushait. J Family Med Prim Care. 2020;9(1):99–104. 10.4103/jfmpc.jfmpc_530_19 PMC7014839 32110573

[32] Zhou X, Li J, Deng S, Xu Z, Liu Z. Ropivacaine at different concentrations on intrapartum fever, IL-6 and TNF-α in parturient with epidural labor analgesia. Exp Ther Med. 2019;17(3):1631–6. 30783430 10.3892/etm.2018.7121 PMC6364190

[33] Tian F, Wang K, Hu J, Xie Y, Sun S, Zou Z, Huang S. Continuous spinal anesthesia with sufentanil in labor analgesia can induce maternal febrile responses in puerperas. Int J Clin Exp Med. 2013;6(5):334–41. 23724151 PMC3663999

[34] Armstrong S, Fernando R. Side effects and efficacy of neuraxial opioids in pregnant patients at delivery: a comprehensive review. Drug Saf. 2016;39(5):381–99. 26832926 10.1007/s40264-015-0386-5

[35] Grant EN, Tao W, Craig M, McIntire D, Leveno K. Neuraxial analgesia effects on labour progression: facts, fallacies, uncertainties and the future. BJOG. 2015;122(3):288–93. 25088476 10.1111/1471-0528.12966 PMC4308552

[36] Sultan P, Murphy C, Halpern S, Carvalho B. The effect of low concentrations versus high concentrations of local anesthetics for labour analgesia on obstetric and anesthetic outcomes: a meta-analysis. Can J Anaesth. 2013;60(9):840–54. 23925722 10.1007/s12630-013-9981-z

[37] Hawker L, Weeks A. Postpartum haemorrhage (PPH) rates in randomized trials of PPH prophylactic interventions and the effect of underlying participant PPH risk: a meta-analysis. BMC Pregnancy Childbirth. 2020;20(1):107. 32054453 10.1186/s12884-020-2719-3 PMC7020586

[38] Xiao J, Yi W, Wu L. Effects of electroacupuncture on reducing labor pain and complications in the labor analgesia process of combined spinal-epidural analgesia with patient-controlled epidural analgesia. Arch Gynecol Obstet. 2019;299(1):123–8. 30426192 10.1007/s00404-018-4955-6

[39] Vernon TJ, Vogel TM, Dalby PL, Mandell G, Lim G. Ultrasound-assisted epidural labor analgesia for landmark identification in morbidly obese pregnant women: a preliminary investigation. J Clin Anesth. 2020;59:53–4. 31226533 10.1016/j.jclinane.2019.05.023 PMC6851414

[40] Junge C, von Soest T, Weidner K, Seidler A, Eberhard-Gran M, Garthus-Niegel S. Labor pain in women with and without severe fear of childbirth: a population-based, longitudinal study. Birth. 2018;45(4):469–77. 29630751 10.1111/birt.12349

